# Sample Size Calculations for Stepped Wedge Designs with Treatment Effects that May Change with the Duration of Time under Intervention

**DOI:** 10.1007/s11121-023-01587-1

**Published:** 2023-09-20

**Authors:** James P. Hughes, Wen-Yu Lee, Andrea B. Troxel, Patrick J. Heagerty

**Affiliations:** 1 Department of Biostatistics, University of Washington, Seattle, WA 98195 USA; 2 Department of Population Health, Division of Biostatistics, New York University, New York, NY USA

**Keywords:** Sample size, Stepped wedge, Exposure time indicator model

## Abstract

The stepped wedge design is often used to evaluate interventions as they are rolled out across schools, health clinics, communities, or other clusters. Most models used in the design and analysis of stepped wedge trials assume that the intervention effect is immediate and constant over time following implementation of the intervention (the “exposure time”). This is known as the IT (immediate treatment effect) assumption. However, recent research has shown that using methods based on the IT assumption when the treatment effect varies over exposure time can give extremely misleading results. In this manuscript, we discuss the need to carefully specify an appropriate measure of the treatment effect when the IT assumption is violated and we show how a stepped wedge trial can be powered when it is anticipated that the treatment effect will vary as a function of the exposure time. Specifically, we describe how to power a trial when the exposure time indicator (ETI) model of Kenny et al. (Statistics in Medicine, 41, 4311–4339, 2022) is used and the estimand of interest is a weighted average of the time-varying treatment effects. We apply these methods to the ADDRESS-BP trial, a type 3 hybrid implementation study designed to address racial disparities in health care by evaluating a practice-based implementation strategy to reduce hypertension in African American communities.

**Supplementary Information:**

The online version contains supplementary material available at 10.1007/s11121-023-01587-1.

## Introduction

In prevention research, clinical research, and implementation science, the stepped wedge design is often used to evaluate interventions as they are rolled out across schools, health clinics, communities, or other clusters (Hussey & Hughes, 2007; Copas et al., 2015; Hemming et al., 2017; Hemming et al., 2018). In a stepped wedge design, all clusters typically start in a control or standard of care condition and then, at pre-selected intervals, the intervention is introduced in one or more randomly selected clusters, until all clusters receive the intervention. Outcome measurements are usually collected in each cluster in each interval, although other designs are possible (Hooper & Burke, 2015; Kasza et al., 2022). A key motivation for the use of the stepped wedge design is to study alternative implementation strategies, tailored to the local context, for evidence-based interventions.

Most analyses of data from stepped wedge trials assume that the intervention effect is “instantaneous” (reaches full effect within the time interval in which it is introduced) and constant (does not vary as a function of time since introduction—the “exposure time”). Most papers on sample size calculations for stepped wedge designs (Hemming & Taljaard, 2016; Hooper et al., 2016; Xia et al., 2021) and software for sample size calculations for stepped wedge designs incorporate this instantaneous treatment (IT) effect assumption. Some of these programs also allow one to prespecify a transition period or pre-specified fractional treatment effects (Hughes et al., 2015) and some allow for a linearly increasing or decreasing treatment time effects (see Ouyang et al., 2022 for a review of packages).

More generally, however, the intervention effect may vary as an arbitrary function of exposure time. Importantly, Kenny et al. (2022) show that assuming the intervention effect is instantaneous and constant; when it is not can lead to extremely misleading estimates of the intervention effect (see, for example, Fig. 2 in Kenny et al. (2022)). As an alternative, Kenny et al. (2022) propose an “exposure time indicator” (ETI) model for analysis of stepped wedge trials. In this manuscript, we recap the ETI model of Kenny et al. (2022) and illustrate how one can do sample size calculations for various estimands under an ETI model using the R software package, swCRTdesign. 

Our motivating example is the ADDRESS-BP trial, a type 3 hybrid implementation study. The ADDRESS-BP study addresses a critical health issue: Black adults have the highest rate of hypertension in the USA, nearly twice the risk of fatal stroke (Egan et al., 2010; Centers for Disease Control and Prevention, 2000), and a 50% higher rate of cardiovascular disease mortality (Giles et al., 1995; Klag et al., 1997; Pavlik et al., 1997; Singh et al., 1996) compared to the general population (Gyamfi et al., 2022). Barriers to hypertension control exist at multiple levels, including the patient (e.g., poor adherence and lack of patient engagement), the physician (e.g., clinical inertia), and the health system (e.g., poor integration of clinical decision support tools into care). ADDRESS-BP is designed to evaluate a novel implementation strategy to reduce hypertension in African Americans and address racial disparities in cardiovascular and pulmonary health, disease, and disease risk factors in high-burden communities in the USA.

**Fig. 1:**
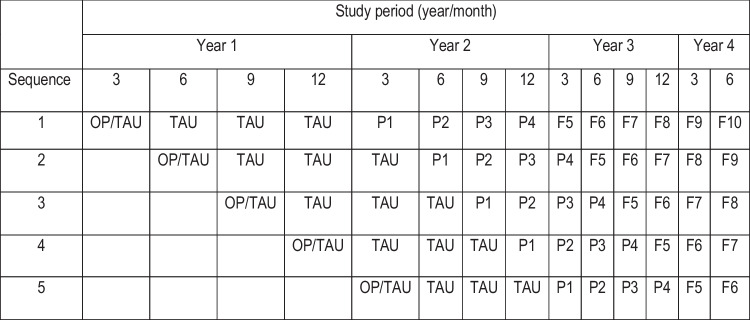
ADDRESS-BP trial design. Each row corresponds to a sequence and there are five health care facilities per sequence. No data are collected in empty cells. In each sequence there are 4 “control” periods (OP, on-boarding period; TAU, training as usual) and up to 10 “exposed” periods (i.e., up to 10 exposure times). Exposure to the PATCH implementation strategy begins at exposure time P1 and there is scientific interest in assessing both the short-term (P#, PATCH implementation strategy at exposure time #) and long-term (F#, follow-up periods at exposure time #) effects of the strategy

The ADDRESS-BP study uses a stepped-wedge design (see Fig. 1) with five sequences and 14 periods to evaluate PATCH, a tailored practice facilitation, and community health worker implementation strategy designed to promote adoption of an evidence-based intervention (Practice support and Community Engagement—PACE) for the treatment of uncontrolled hypertension. PACE is delivered to patients by nurse case managers and community health workers, who address patients’ social risk factors (described as “specific adverse social conditions that are associated with poor health”) for management of chronic conditions, including hypertension. The PATCH implementation strategy will be compared to a training-as-usual (TAU) implementation strategy; key outcomes are facility adoption of PATCH and blood pressure control by individuals treated at these facilities.

In this manuscript we first review the statistical methodology necessary to conduct power and sample size evaluation for stepped-wedge designs similar to ADDRESS-BP. We then illustrate the use of the developed tools using the ADDRESS-BP design with a goal of facilitating the use of these methods for future prevention and intervention studies.

## Methodology

Let ${Y}_{ijk}$ represent the outcome measurement for person *k* (*k = 1 ,…, m*_*ij*_) in cluster *i* (*i = 1 ,…, I*) at time period *j* (*j = 1 ,…, J*) (Table 1 summarizes the notation used in this section). Mixed effects models are convenient for sample size calculations in stepped-wedge trials as they allow one to explicitly specify a correlation structure for the observations (Xia et al., 2021). Therefore, we use the following general model for analysis of stepped wedge design trials:

$$g\left({\mu }_{ijk}|{b}_{ij}\right)={X}_{ij}\beta +{Z}_{ij}{b}_{ij}$$

where ${\mu }_{ijk}$ is the mean of ${Y}_{ijk}$, *g* is a link function, and ${X}_{ij}$ is a design matrix for the fixed effects, $\beta ={\left(\Gamma ,\delta \right)}^{T}$, which are partitioned into a vector of parameters capturing the underlying trend in study time ($\Gamma$) and a scalar or vector of parameters capturing the intervention effect ($\delta$) (more on this below). Finally, we use ${Z}_{ij}$ (a design matrix) and ${b}_{i\bullet }\sim N(0,D)$ (a vector of random effects) to quantify the correlation among observations in cluster *i*. Random effects commonly included in the analysis of stepped-wedge trials include cluster, cluster by time, cluster by treatment, and, for cohort designs, an individual random effect (Hooper et al., 2016). Decaying auto-correlation structures have also been proposed, although these cannot be represented with random effects (Kasza et al., 2017). Li et al (2021) provide a comprehensive overview of the mixed-effect model framework for stepped-wedge designs. Ouyang et al. (2023) show the relationship between the random effects parameterization given above and the correlation coefficient (i.e., intra-cluster correlations) parameterization for specifying dependence in the data.

**Table 1:** Notation

Notation	Description
${Y}_{ijk}$	outcome measurement for person *k* (*k = 1 ,…, m*_*ij*_) in cluster *i* (*i = 1 ,…, I*) at time *j* (*j = 1 ,…, J*)
${\mu }_{ijk}$	mean of ${Y}_{ijk}$
*g( )*	link function in the generalized linear models framework
${X}_{ij}$	design matrix for the fixed effects $\beta$
${Z}_{ij}$	design matrix for the random effects ${b}_{i\bullet }$
$\beta ={\left(\Gamma ,\delta \right)}^{T}$	$\beta$ is a vector of parameters composed of a vector of parameters that capture the underlying trend in study time ($\Gamma$) and a scalar ($\delta$) or vector ($\delta =({\delta }_{1},\dots , {\delta }_{S})$) of parameters capturing the intervention effect over *S* exposure times
${b}_{i\bullet }$	Vector of random effects
$H=\left({h}_{1},{h}_{2},\dots ,{h}_{S}\right)$	Weighting vector (with constraint $\sum {h}_{i}=1)$ used with $\delta$ to form the estimand $\Psi$
$\Psi ={\sum }_{s=1}^{S}{h}_{s}{\delta }_{s}$	Summary intervention effect of interest
$\Sigma$	Variance of $\widehat{\beta }$
${\mathrm{Var}}_{0}\left(\widehat{\Psi }\right), {\mathrm{Var}}_{\mathrm{a}}\left(\widehat{\Psi }\right)$	Variance of $\widehat{\Psi }$ under the null and alternative hypotheses, respectively

Hughes et al. (2015) discuss approaches to eliciting variances of the random effects for the purposes of power calculations. For binomial or Poisson endpoints, these variance components are typically expressed on the proportion or count scale, respectively, but may be transformed to the scale corresponding to the link function *g* using the delta method^1^.

Let $Var\left(\widehat{\beta }\right)=\Sigma$. Note that Σ is a function of the study design and the variances of the random effects, quantities that must be specified during the design of the study to determine power. Following Xia et al. (2021), Σ may be expressed as

$$\Sigma ={\left({X}^{T}{V}^{-1}X\right)}^{-1}$$

where

$$V=W+ZD{Z}^{T}.$$

And *W* is a diagonal matrix with entries ${w}_{i}=\phi {a}_{i}\nu \left({\mu }_{i}\right){\left[{g}{^{\prime}}\left({\mu }_{i}\right)\right]}^{2}$ (see Table 2). For a normal distribution, ${w}_{i}= {\sigma }^{2}.$ Σ may be partitioned as

**Table 2:** Variance function values for selected distributions with canonical links

Distribution (link)	*ϕ*	*a*	$\nu (\mu )$	$g(\mu )$	$g{\prime}(\mu )$
Normal (identity)	${\sigma }^{2}$	1	1	$\mu$	1
Bernoulli (logit)	1	1	$\mu (1-\mu )$	$\mathrm{log}\left(\frac{\mu }{1-\mu }\right)$	$\frac{1}{\mu (1-\mu )}$
Poisson (log)	1	$\frac{1}{m}$	$\mu$	$\mathrm{log}(\mu )$	$\frac{1}{\mu }$
Binomial (logit)	1	$\frac{1}{m}$	$\mu (1-\mu )$	$\mathrm{log}\left(\frac{\mu }{1-\mu }\right)$	$\frac{1}{\mu (1-\mu )}$

$$\Sigma =\left(\begin{array}{cc}{\Sigma }_{\mathrm{\Gamma \Gamma }}& {\Sigma }_{\Gamma \delta }\\ {\Sigma }_{\Gamma \delta }^{T}& {\Sigma }_{\delta \delta }\end{array}\right).$$

As noted in the introduction, most models for analysis and sample size calculations assume that the intervention effect,$\delta$, is a scalar and that the corresponding element of ${X}_{ij}$ is a simple 0/1 measure, indicating whether the intervention is turned off or on in cluster *i* at time *j* (this is the IT model mentioned above). Alternatively, Kenny et al. (2022) assume that $\delta =({\delta }_{1},\dots , {\delta }_{S})$, where ${\delta }_{s}$ is the effect of the intervention after a cluster has been exposed to the intervention for *s* time units (exposure time = *s*) (the ETI model) and *S* is the maximum exposure time (note that *S = J* − 1 in a classic stepped wedge design where *J* = the number of sequences + 1).

Under the ETI model, the entire vector δ may be of interest, or the investigator may define a function of δ as a summary of the intervention effect. For example, Kenny et al. (2022) define the time-averaged treatment effect over an exposure interval *s*_1_ to *s*_2_ as

$$\Psi \left[{s}_{1},{s}_{2}\right]\equiv \frac{1}{{s}_{2}-{s}_{1}+1}{\sum }_{r={s}_{1}}^{{s}_{2}}{\delta }_{r}.$$

Alternatively, interest may lie in the treatment effect at a specific time *s* (point treatment effect):

$${\Psi }_{s}= {\delta }_{s}.$$

More generally, most summary estimands of interest can be written as a linear combination of the ${\delta }_{s}$:

1 $$\Psi ={\sum }_{s=1}^{S}{h}_{s}{\delta }_{s}.$$

for known constants *h*_*s*_. Letting $H=\left({h}_{1},{h}_{2},\dots ,{h}_{S}\right)$ (with constraint $\sum {h}_{i}=1)$, $\widehat{\updelta }=({\widehat{\delta }}_{1},{\widehat{\delta }}_{2},\dots , {\widehat{\delta }}_{S})$, and ${\Sigma }_{\mathrm{\delta \delta }}=\mathrm{Var}(\widehat{\updelta })$ we can write the variance of the estimated intervention effect, $\widehat{\Psi }$, as

$$\mathrm{Var}\left(\widehat{\Psi }\right)=H{\Sigma }_{\delta \delta }{H}^{T}.$$

Therefore, given a hypothesized intervention effect (*ψ*), weighting vector (H), pre-specified study design, and variance components, and assuming a two-sided type-1 error rate of *α*, the power of the study can be computed as

2 $$\Phi \left(\frac{\left|\Psi \right|-{\Phi }^{-1}\left(1-{}^{\alpha}/_{2}\right)*\sqrt{{\mathrm{Var}}_{0}\left(\widehat{\Psi }\right)}}{\sqrt{{\mathrm{Var}}_{\mathrm{a}}\left(\widehat{\Psi }\right)}}\right)$$

where Φ is the normal cumulative distribution function and ${\mathrm{Var}}_{0}\left(\widehat{\Psi }\right)$ and ${\mathrm{Var}}_{\mathrm{a}}\left(\widehat{\Psi }\right)$ are the variances under the null and alternative hypotheses, respectively. For the normal distribution with identity link, power depends only on Ψ and not the individual ${\delta }_{s}$ (and ${\mathrm{Var}}_{0}\left(\widehat{\Psi }\right)$ and ${\mathrm{Var}}_{\mathrm{a}}\left(\widehat{\Psi }\right)$ are equal in this case). For non-identity links, power will depend on the individual ${\delta }_{s}$ through ${\mathrm{Var}}_{\mathrm{a}}\left(\widehat{\Psi }\right)$.

The R package swCRTdesign can be used to implement the methods described above for cross-sectional and closed cohort designs (specifically, swCRTdesign implements Eq. (2) which does not, in general, simplify to a design effect (Hooper et al., 2016)). swCRTdesign has two functions for computing study power, swPwr() and swGlmPwr(). swPwr() computes power using an identity link for data with a normal or binomial distribution. swGlmPwr()can compute power for binomial data with a logit link or Poisson data with a log link. Both functions can compute power for either an IT model or an ETI model with user-specified H vector (allowing specification of a time-averaged treatment effect, a point treatment effect, or other summaries) and both can incorporate random effects for cluster, cluster by time, cluster by treatment, and, for cohort designs, individual.

## Data

In our motivating example, ADDRESS-BP, the unit of randomization is the practice facility. There are 25 practice facilities (5 per sequence) and three treatment teams which consist of one practice facilitator and one community health worker per team; each treatment team will work with multiple facilities. During an onboarding period, each facility will establish a closed cohort of approximately 20 individuals with uncontrolled hypertension. Facilities will then provide training as usual (TAU) for the PACE intervention until the PATCH implementation strategy is introduced, as illustrated in Fig. 1.

In this paper we focus on the clinical outcome of blood pressure control (yes/no) in the individual patient. During the TAU periods we expect 40% of participants will achieve blood pressure control. We expect the PATCH implementation strategy may take up to 6 months (2 time periods—each time period is 3 months) to achieve full effect so we propose to evaluate PATCH 6 to 12 months following introduction (exposure times P3 and P4 in Fig. 1). We believe PATCH will improve blood pressure control to 60% during this period. To evaluate the sustainability of this implementation strategy, we will also estimate the proportion of patients achieving/maintaining blood pressure control in exposure times 5–10 following PATCH introduction (the follow-up period—F5–F10 in Fig. 1).

Preliminary data are available from another NYU study, Advancing Medication Adherence for Latinos with Hypertension through a Team-based Care Approach, that also utilized a stepped-wedge cluster randomized design and practice facilitation to improve blood pressure control (Schoenthaler et al., 2021); these data enabled estimation of variance components for practice facility, practice facility by time, and individual. As noted above, each treatment team will serve multiple facilities and may represent an additional source of variation. However, due to software limitations, we were unable to include that source of variation in our power calculations.

## Results

The R package swCRTdesign was used to compute power for the ADDRESS-BP study. Table 3 lists parameter values for the power calculations. R code for each result given below is included in the Appendix and referenced below as R# (e.g., R1, R2, etc.). Note that all the results from swGlmPwr (R1–R5) have been verified by simulation.

**Table 3:** Parameter values used for power calculations for ADDRESS-BP trial. See Fig. 1 for definition of TAU, P3, P4

Parameter description	Value	
Number of observations per facility per time period	0 pre-onboarding; otherwise, *n* = 20	
Outcome percent during TAU period	40%	
Outcome percent during exposure times three (P3) and four (P4)	60%	
Time trend (on logit scale)	0.08/period	
	Variance (on logit scale)	Intra-cluster correlation^a^
Cluster variance/between-period ICC	0.1316	0.022
Cluster*time variance/within-period ICC	0.1974	0.054
Individual variance/within-individual ICC	2.5	0.430

*ICC* intra-cluster correlation

^a^ICCs are expressed on logit scale with *σ*^2^ = *π*^2^/3 ≈ 3.290 (Eldridge et al., 2009). See Ouyang et al. (2023) table 1 for definitions of ICCs

**Fig. 2:**
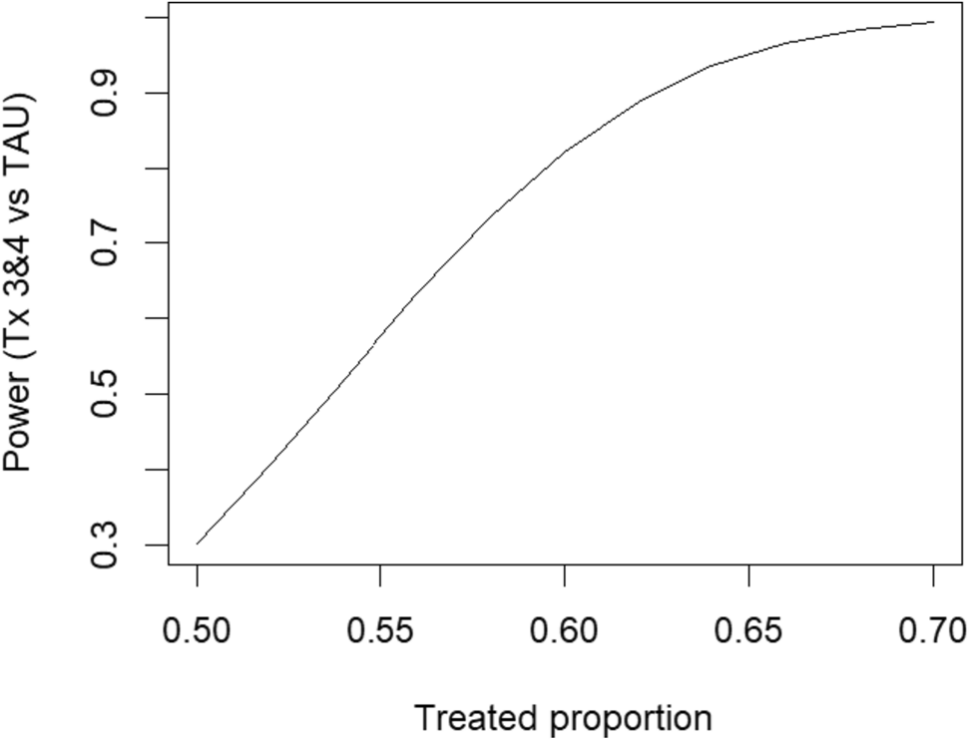
Power for comparing treatment exposure times 3 and 4 versus TAU. Assumed proportion during TAU is 0.40

Figure 2 shows the power comparing the primary outcome of interest, the average blood pressure control over exposure times 3 and 4, shown as P3 and P4 in Fig. 1, versus TAU based on the ETI model of Kenny et al. (2022) (R1). Since the maximum number of exposure time periods is 10 (P1–F10 in Fig. 1), this comparison corresponds to simply averaging the responses in periods P3 and P4 (i.e., *H* = (0, 0, 0.5, 0.5, 0, 0, 0, 0, 0, 0) in Eq. (1)). For the alternative of 60% of patients achieving blood pressure control, the power is 82% (R2). For comparison, an IT model that compares all PATCH periods to TAU would have power 99.9% (R3) although, as noted above, this model is susceptible to substantial bias and possible loss of power if the IT assumption is wrong.

Note that the power analysis shown above assumes that no data are available for cohort members prior to the onboarding period (Fig. 1). An alternative would be to form the cohorts in all clusters at the start of the study (start of year 1) so that data were available from all clusters in all time periods. In this case, the power for comparing exposure times 3 and 4 to TAU would increase to 92% (R4).

Interestingly, the power for comparing the average treatment effect over the follow-up period (exposure times F5–F10) versus TAU under the same alternative is only 39% (R5). Even though there is more data in the follow-up period, power is lower because (i) there are fewer cluster periods at later exposure times and (ii) there are no direct vertical (between cluster) comparisons between treatment and control in exposure times 5 through 10.

The ETI model is inefficient for estimating the treatment effect compared to the IT model when the treatment effect is constant. To recover some of the efficiency, but still reduce the risk of bias, one could assume a piecewise constant treatment effect (this is effectively a version of the spline approach discussed by Kenny et al. (2022)). If we assume piecewise constant treatment effects for exposure times P1–P2, exposure times P3–P4, and exposure times F5–F10 then the power to compare exposure times P3–P4 versus TAU is 94% and to compare exposure times F5–F10 to TAU is 75% (R6). Note that including data from exposure times P1 and P2 in the analysis improves power by contributing to estimation of the temporal trend component of the model, even though these periods are not part of the primary or secondary hypotheses.

## Discussion

We have presented a set of generalizable tools for designing stepped-wedge studies that can be used to evaluate the impact of interventions during real-world implementation, and we have demonstrated their use in the ADDRESS-BP study, a type 3 hybrid implementation study designed to address racial disparities in health care by evaluating a novel implementation strategy for a practice-based intervention to reduce hypertension in African American communities.

The ETI model of Kenny et al. (2022) relaxes the assumption of an immediate and constant response to treatment in step-wedge studies and allows the treatment effect to vary as a function of exposure time. In this article we have shown how power calculations for analysis of a stepped-wedge trial based on the ETI model can be conducted. The package, swCRTdesign, can be used to implement these calculations. Importantly, when the treatment effect varies as a function of exposure time, careful scientific thought must be used to define the estimand of interest and this choice can strongly influence power. This effectively means defining the weighting vector H (and the intervention effect size associated with H). However, this is the only additional requirement needed for sample size calculations for the ETI model compared to the traditional IT model. An alternative approach is discussed by Maleyeff et al. (2022) in which they assume that the treatment effect varies randomly over exposure time; they then provide power calculations for estimating the average treatment effect.

It is at present unclear how often the treatment effect varies significantly with exposure time but Kenny et al. (2022) recommend that the IT model should not be used unless the assumption of an immediate and constant treatment effect is justifiable based on contextual knowledge of the intervention. They also note a need to reanalyze data from past stepped-wedge trials to understand the prevalence of time-varying treatment effects.

ETI-based estimators are less efficient than estimates based on an IT model but are robust against the assumption of a constant treatment effect, violation of which can lead to an extremely misleading treatment effect estimate. One approach to recovering some efficiency and improving power is to model the treatment effect using a piecewise constant or higher-order spline function. The piecewise constant approach can be particularly useful when the period of interest corresponds directly to one of the constant pieces of the spline. However, whenever one assumes that the treatment is constant over multiple exposure times, there is a potential for the type of misleading treatment estimates observed by Kenny et al. (2022). In the case of the ADDRESS-BP trial, we believe it is unlikely that combining exposure times P3 and P4 would lead to any problems because (i) only two adjacent periods are being combined and (ii) those two periods have similar information content. In contrast, there is a greater risk in assuming that the treatment effect is constant over the entire follow-up period, i.e., exposure times F5–F10.

We have also seen in the ADDRESS-BP case study that, under the ETI model, power is greater for studying the treatment effect immediately after the transition from control to treatment compared to treatment effects at longer exposure times, consistent with Kasza and Forbes (2019). We also noted in the ADDRESS-BP trial that including data that does not directly inform the treatment effect estimate of interest can improve power by providing more precise estimation of other model parameters. More research is needed, however, to fully understand how other modeling choices (e.g., using spline-based models for the underlying time trend and/or the exposure time trend, extending follow-up, etc.) affect power for testing short-term and long-term treatment effects.

Interestingly, because of the partial collinearity between study time and treatment, as well as the complex correlation structure in the stepped-wedge design, standard statistical intuition regarding power may be misleading. For example, in the ADDRESS-BP study power analysis, the power for comparing exposure times P3 and P4 to TAU is greater than the power for comparing exposure times F5–F10 to TAU even though there is quantitatively more data for the latter comparison. This is a result of several interacting factors: (i) decreasing information (and, therefore, greater variation) for exposure times F7 and above; (ii) the lack of a between-cluster comparison between exposure times F5–F10 and TAU; (iii) the correlation structure of the model which affects the relative information provided by between-cluster versus within-cluster comparisons; and (iv) the need to estimate study time effects, which are partly collinear with treatment.

One limitation of the power analysis of the ADDRESS-BP study presented above is that we have not accounted for the effect of the treatment teams, each of which will work across multiple facilities. Davis-Plourde et al. (2023) describe sample size calculations for a multi-level stepped-wedge study with nested clusters (in this case, facilities nested within treatment teams) and their idea of adding additional random effects to account for additional levels of clustering could be used with both the IT and ETI models. However, due to software limitations, we are not able to account for multiple levels of clustering in this analysis although some insight is possible by including a nonzero eta argument in calls to swGlmPwr. eta characterizes the variation in the treatment effect between facilities (whereas, ideally, we would like to include variation in the treatment effect between treatment teams—see Brown et al., 2022). We found that power generally declines as eta increases, suggesting that inclusion of a treatment team effect may decrease power modestly. The effect of treatment teams should be included in the final analysis of the ADDRESS-BP data.

The ADDRESS-BP trial will evaluate a multi-component implementation strategy leveraging practice facilitation, nurse case managers, and community health workers to promote implementation of home blood pressure monitoring and incorporation of social determinants of health to improve blood pressure control (Odedosu et al., 2012; Pickering et al., 2008). The study will use the ETI model and is well powered to address a clinically meaningful increase in rate of blood pressure control at 12 months following initiation of PATCH.

## Supplementary Information

Below is the link to the electronic supplementary material.

Supplementary file1 (DOCX 40 KB)

## Footnotes

1 Specifically, $Va{r}_{g(x)}={Var}_{x} {g}{\prime}{\left(x\right)}^{2}$. For example, for a binomial endpoint on the logit scale, we would apply the transformation $Va{r}_{logit(p)}=\frac{Va{r}_{p}}{{p}^{2}{\left(1-p\right)}^{2}}$ to random effect variances expressed on the proportion scale.
