# Correction: Sample Size Calculations for Stepped Wedge Designs with Treatment Effects that May Change with the Duration of Time under Intervention

**DOI:** 10.1007/s11121-024-01645-2

**Published:** 2024-01-06

**Authors:** James P. Hughes, Wen-Yu Lee, Andrea B. Troxel, Patrick J. Heagerty

**Affiliations:** 1 Department of Biostatistics, University of Washington, Seattle, WA 98195 USA; 2 Department of Population Health, Division of Biostatistics, New York University, New York, NY USA

**Correction to: Prevention Science** 10.1007/s11121-023-01587-1

The article “Sample Size Calculations for Stepped Wedge Designs with Treatment Effects that May Change with the Duration of Time under Intervention”, written by Hughes, J.P., Lee, W-Y., Troxel, A.B., and Heagerty, P.J., was originally published electronically on the publisher’s internet portal on 20 September 2023 without open access. With the author(s)’ decision to opt for Open Choice the copyright of the article changed on 13 December 2023 to © The Author(s) 2023 and the article is forthwith distributed under a Creative Commons Attribution 4.0 International License, which permits use, sharing, adaptation, distribution and reproduction in any medium or format, as long as you give appropriate credit to the original author(s) and the source, provide a link to the Creative Commons licence, and indicate if changes were made. The images or other third party material in this article are included in the article’s Creative Commons licence, unless indicated otherwise in a credit line to the material. If material is not included in the article’s Creative Commons licence and your intended use is not permitted by statutory regulation or exceeds the permitted use, you will need to obtain permission directly from the copyright holder. To view a copy of this licence, visit http://creativecommons.org/licenses/by/4.0/.

The original article has been corrected.

