# Correction: Sample Size Calculations for Stepped Wedge Designs with Treatment Effects that May Change with the Duration of Time under Intervention

**DOI:** 10.1007/s11121-024-01652-3

**Published:** 2024-02-16

**Authors:** James P. Hughes, Wen-Yu Lee, Andrea B. Troxel, Patrick J. Heagerty

**Affiliations:** 1 Department of Biostatistics, University of Washington, Seattle, WA 98195 USA; 2 Department of Population Health, Division of Biostatistics, New York University, New York, NY USA

**Correction to: Prevention Science** 10.1007/s11121-023-01587-1

In this article, the Funding information section was updated and should have read ‘This research was supported by NIH grants AI29168, UG3HL151310-01, and UH3HL151310-04.’

The original article has been corrected.

