# Supplementary material for: Sample Size Calculations for Stepped Wedge Designs with Treatment Effects that May Change with the Duration of Time under Intervention
Source: Prev Sci. 2023 Sep 20;25(Suppl 3):348–55. doi: 10.1007/s11121-023-01587-1 (PMC10950842; doi:10.1007/s11121-023-01587-1)
Supplement: Supplementary file 1 — Supplementary file1 (DOCX 40 KB) [file 11121_2023_1587_MOESM1_ESM.docx]

Appendix

# Preliminaries

library(swCRTdesign)

#version 4.0

logit <- function(x){log(x/(1 - x))}

expit <- function(x){exp(x)/(1 + exp(x))}

sample_size = matrix(c(rep(c(20,20,20,20,20,20,20,20,20,20,20,20,20,20),5),

rep(c( 0,20,20,20,20,20,20,20,20,20,20,20,20,20),5),

rep(c( 0, 0,20,20,20,20,20,20,20,20,20,20,20,20),5),

rep(c( 0, 0, 0,20,20,20,20,20,20,20,20,20,20,20),5),

rep(c( 0, 0, 0, 0,20,20,20,20,20,20,20,20,20,20),5)),

25,14,byrow=TRUE)

design1=swDsn(c(5,5,5,5,5),extra.ctrl.time=3,extra.trt.time=5)

design2=swDsn(c(5,5,5,5,5),

swBlk= matrix(c(0,0,0,0,1,1,2,2,3,3,3,3,3,3,

NA,0,0,0,0,1,1,2,2,3,3,3,3,3,

NA,NA,0,0,0,0,1,1,2,2,3,3,3,3,

NA,NA,NA,0,0,0,0,1,1,2,2,3,3,3,

NA,NA,NA,NA,0,0,0,0,1,1,2,2,3,3),

5,14,byrow=TRUE))

#R1

tx = seq(0.5,0.7,.02)

pwr = rep(NA,length(tx))

for (i in 1:length(tx)){

pwr[i] = swGlmPwr(design1,distn="binomial", n=sample_size,

fixed.intercept=logit(0.40),

fixed.treatment.effect=c(0,0,rep(logit(tx[i])-logit(0.40),8)),

fixed.time.effect=0.08,

H=c(0,0,1,1,0,0,0,0,0,0),

tau=sqrt(.1316),gamma=sqrt(.1974),eta=0,zeta=sqrt(2.5))

# note that H is automatically normalize to sum to 1.0 within swGlmPwr

}

plot(tx,pwr,xlab="Treated proportion (TAU = 0.40)",

ylab="Power (Tx 3&4 vs TAU)",type="l")

#R2

swGlmPwr(design1,distn="binomial", n=sample_size,

fixed.intercept=logit(0.40),

fixed.treatment.effect=c(0,0,rep(logit(0.60)-logit(0.40),8)),

fixed.time.effect=0.08,

H=c(0,0,1,1,0,0,0,0,0,0),

tau=sqrt(.1316),gamma=sqrt(.1974),eta=0,zeta=sqrt(2.5))

# R3

swGlmPwr(design1,distn="binomial",n=sample_size,

fixed.intercept=logit(0.40),

fixed.treatment.effect=logit(0.60)-logit(0.40),

fixed.time.effect=0.08,

tau=sqrt(.1316),gamma=sqrt(.1974),eta=0,zeta=sqrt(2.5))

# R4

swGlmPwr(design1,distn="binomial",n=20,

fixed.intercept=logit(0.40),

fixed.treatment.effect=c(0,0,rep(logit(0.60)-logit(0.40),8)),

fixed.time.effect=0.08,

H=c(0,0,1,1,0,0,0,0,0,0),

tau=sqrt(.1316),gamma=sqrt(.1974),eta=0,zeta=sqrt(2.5))

# R5

swGlmPwr(design1,distn="binomial",n=sample_size,

fixed.intercept=logit(0.40),

fixed.treatment.effect=c(0,0,rep(logit(0.60)-logit(0.40),8)),

fixed.time.effect=0.08,H=c(0,0,0,0,1,1,1,1,1,1),

tau=sqrt(.1316),gamma=sqrt(.1974),eta=0,zeta=sqrt(2.5))

# R6

swGlmPwr(design2,distn="binomial",n=20,

fixed.intercept=logit(0.40),

fixed.treatment.effect=c(0,rep(logit(0.60)-logit(0.40),2)),

fixed.time.effect=0.08,

tau=sqrt(.1316),gamma=sqrt(.1974),eta=0,zeta=sqrt(2.5))
